# A large-scale fMRI dataset for the visual processing of naturalistic scenes

**DOI:** 10.1038/s41597-023-02471-x

**Published:** 2023-08-23

**Authors:** Zhengxin Gong, Ming Zhou, Yuxuan Dai, Yushan Wen, Youyi Liu, Zonglei Zhen

**Affiliations:** 1 Beijing Key Laboratory of Applied Experimental Psychology, Faculty of Psychology, Beijing Normal University, Beijing, 100875 China; 2 State Key Laboratory of Cognitive Neuroscience and Learning & IDG/McGovern Institute for Brain Research, Beijing Normal University, Beijing, 100875 China

**Keywords:** Perception, Neural encoding

## Abstract

One ultimate goal of visual neuroscience is to understand how the brain processes visual stimuli encountered in the natural environment. Achieving this goal requires records of brain responses under massive amounts of naturalistic stimuli. Although the scientific community has put a lot of effort into collecting large-scale functional magnetic resonance imaging (fMRI) data under naturalistic stimuli, more naturalistic fMRI datasets are still urgently needed. We present here the Natural Object Dataset (NOD), a large-scale fMRI dataset containing responses to 57,120 naturalistic images from 30 participants. NOD strives for a balance between sampling variation between individuals and sampling variation between stimuli. This enables NOD to be utilized not only for determining whether an observation is generalizable across many individuals, but also for testing whether a response pattern is generalized to a variety of naturalistic stimuli. We anticipate that the NOD together with existing naturalistic neuroimaging datasets will serve as a new impetus for our understanding of the visual processing of naturalistic stimuli.

## Background & Summary

Understanding the neural mechanism of visual processing is an important goal of visual neuroscience. To date, scientists have mostly employed designed artificial stimuli to characterize the response properties of the visual cortex^1–10^. While these controlled stimuli paradigms have yielded much important knowledge on the neural mechanism of visual processing for simple stimuli, challenges remain in understanding how the brain functions to more complex, naturalistic stimuli in real-world environments^4,11–15^. To this end, naturalistic paradigms, which aim to probe the visual system using complex stimuli from the real world, have become increasingly popular^16–20^. However, as the stimuli become increasingly naturalistic, large amounts of neural data are required to effectively characterize the mapping between the stimuli and the neural responses.

Functional magnetic resonance imaging (fMRI) is commonly employed to measure brain activity in studying the neural basis of human visual processing. The cognitive neuroscience community has made significant efforts to collect large-scale fMRI data for naturalistic visual stimuli and make these datasets publicly available^15,17,21–33^. Multiple groups have obtained fMRI datasets using movies as stimuli which contain rich and diverse real-world scenes^23–28^. These datasets have been widely used to investigate the functional organization of the human visual system^34–36^ and test the correspondence between biological and artificial visual systems^37,38^. However, these datasets were not acquired specifically to understand the neural basis of human visual processing. The continuous movie stimulus and the lack of annotations for each frame make them unsuitable for testing specific hypotheses of visual processing. To our knowledge, there are only three large-scale fMRI datasets that have been specifically collected for the study of the neural basis of human visual processing under naturalistic scenes: the BOLD5000 dataset^31^, the Natural Scene Dataset (NSD)^32^, and the THINGS dataset^33^. Notably, the naturalistic images used in the three datasets are selected from the most commonly-used computer vision datasets, including ImageNet^39^, Common Objects in Context (COCO)^40^, Scene UNderstanding (SUN)^41^, and THINGS^42^. The BOLD5000 dataset features 5,000 real-world images as stimuli, scanning four participants with a slow event-related design. The NSD dataset consists of high-resolution 7 T fMRI responses to tens of thousands of naturalistic images, measured with a rapid event-related experiment on eight participants. The THINGS dataset records both fMRI and magnetoencephalographic (MEG) responses to more than 8,000 unique images on three participants with a rapid event-related design. The three unique fMRI datasets have profoundly advanced our understanding of the neural basis of human visual processing in real-world scenes^43–49^. Nonetheless, additional fMRI datasets acquired with naturalistic stimuli are still urgently needed for characterizing both the properties of complex naturalistic stimuli and the response properties of the visual cortex under natural stimulation.

To meet the challenge, we here present another large-scale fMRI dataset called Natural Object Dataset (NOD), which recorded fMRI responses to 57,120 naturalistic images on 30 participants using a rapid event-related paradigm. In the same vein as BOLD5000 and NSD, NOD uses real-world images from the richly annotated ImageNet and COCO databases as stimuli. Moreover, NOD aims to achieve a good balance between sampling variation across individuals and sampling variation across stimuli. On one hand, each of the 30 participants completed an ImageNet scan session with 1,000 different images selected from the ImageNet database to sample variation across individuals as much as possible. On the other hand, nine of 30 participants completed three additional ImageNet sessions with different images to sample variation across stimuli as much as possible. Additionally, one functional localizer session and one COCO session were conducted on these nine participants to enable precision mapping of individual brain activity patterns. The functional localizer session included retinotopic mapping^50^ and category-selective localizer^51^, and the COCO session consisted of 120 COCO images presented ten times. Consequently, the NOD dataset allows researchers to map brain activity patterns within an individual and across participants, as well as to test how a specific visual response is generalized across both stimuli and participants.

This paper provides a comprehensive description of the design, acquisition, and preprocessing of the NOD dataset. We validated the quality of our data via both within-subject and between-subject analyses and illustrated the capacity of the data by building an encoding model to show the hierarchical correspondences between the brain and a deep convolutional neural network (DCNN)^52–55^.

## Methods

### Participants

The Institutional Review Board of Beijing Normal University approved the study (approval number: ICBIR_A_0111_001_02). Flyers approved by the IRB were posted on the campus network to recruit potential participants. Initially, 34 participants were admitted into the experiment. All participants had a normal or corrected-to-normal vision, reported no history of psychiatric or neurological disorders, and provided written informed consent prior to their participation. Four participants were excluded from the NOD experiment because they felt the scan time was too long for them or felt bored with the task in an initial behavior screening session. As a result, a total of 30 healthy participants (18 females), ranging in age between 18 and 26 years (mean ± standard deviation [SD], 21.23 ± 1.96 years), participated in the NOD fMRI experiment. Of these, nine participants were invited to participate in the repeated ImageNet experiment according to their willingness and availability to the multiple scan sessions. All participants provided their informed consent for sharing the anonymized data.

### Experimental design

#### General design

**Fig. 1:**
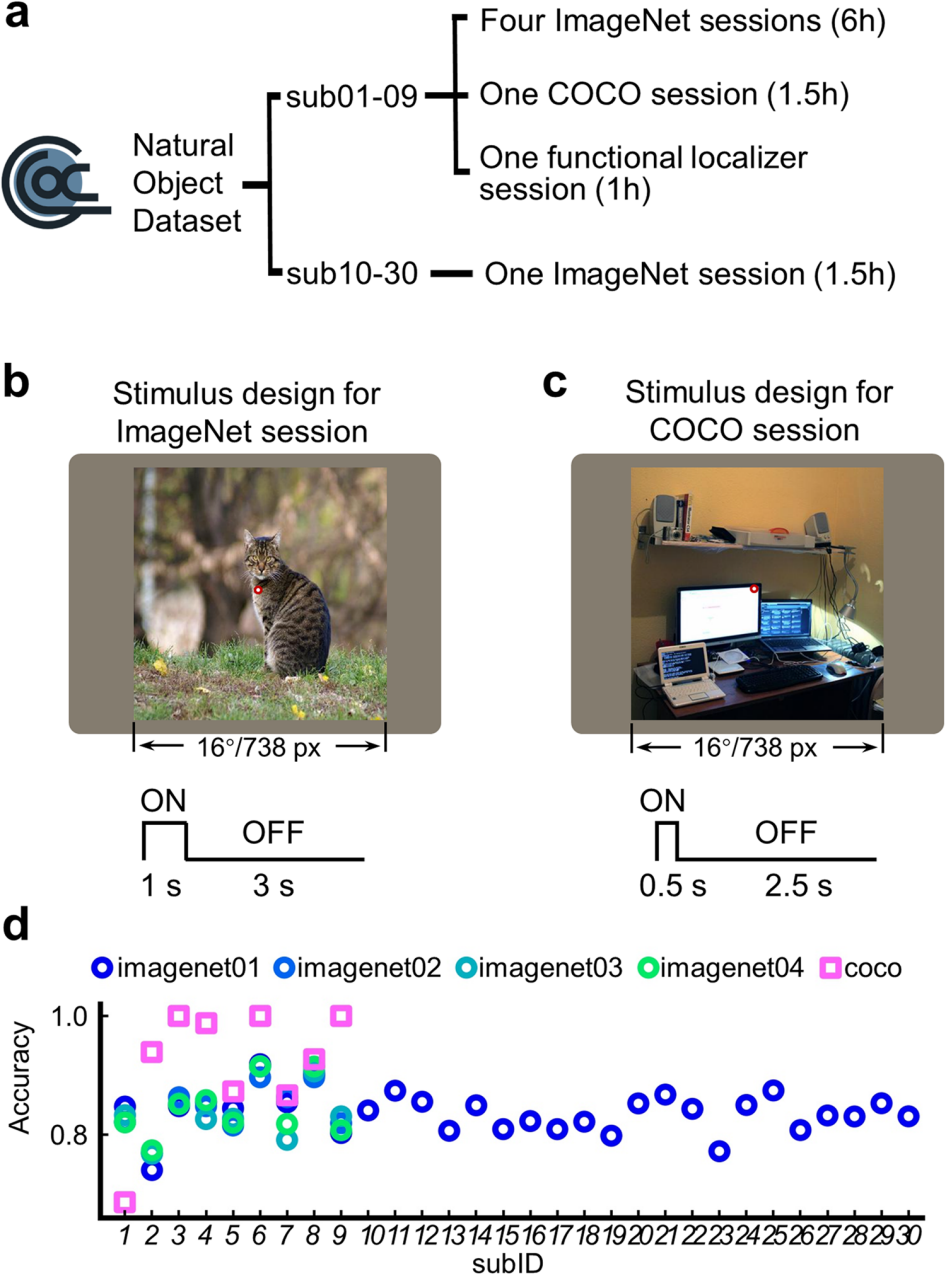
Design of the Natural Object Dataset (NOD) experiments. (**a**) The general design of the NOD experiments. (**b**) The stimulus and trial design for the ImageNet experiment. (**c**) The stimulus and trial design for the COCO experiment. The images selected from the ImageNet and the COCO dataset were resized into square shapes and displayed on a gray background. (**d**) The mean recognition accuracy of each subject in each session of ImageNet and COCO experiments.

The NOD experiment consists of three types of fMRI sessions: ImageNet, COCO, and functional localizer. Each of the 30 participants accomplished an ImageNet session. Among them, nine participants (sub01-09) completed three additional ImageNet sessions, one COCO session, and one functional localizer session (Fig. 1a). An ImageNet session consisted of ten runs, each lasting 8 minutes and 32 seconds. The COCO session consisted of ten runs, each lasting 8 minutes and 2 seconds. The functional localizer session included eight runs for retinotopic mapping (5 minutes /run) and four runs for the category-selective localizer (5 minutes/run). In summary, there are approximately 8.5 hours of MRI scanning for each of the nine participants with multiple sessions and 2 hours of MRI scanning for the twenty-one participants with one session, respectively. All stimuli were presented using Psychophysics Toolbox Version 3 (PTB-3)^56^ via an MR-compatible LCD monitor mounted at the head end of the scanner bore. The monitor operated at a resolution of 1024 pixels × 768 pixels at 60 Hz. The size of the full monitor image was 39.0 cm (width) × 29.5 cm (height). Participants viewed the monitor image through a mirror mounted on the head coil. The viewing distance was around 100 cm (85.5 cm from the mirror to the monitor image + 14.5 cm from the participants’ eyes to the mirror). All stimuli from the various experiments were adjusted to fill 16° of visual angle (738 pixels × 731 pixels).

#### Stimuli from ImageNet

ImageNet is a large-scale visual database designed for computer vision^39^. We chose ImageNet as our core stimuli set for two primary reasons. First, the ImageNet images are naturalistic, but not very complicated. Most objects are centered in the image distinguishable from the background. Second, ImageNet offers diverse semantic annotations for objects organized according to the WordNet hierarchy^57^. In particular, the candidate stimuli set was selected from ImageNet Large Scale Visual Recognition Challenge 2012 (ILSVRC2012), which contains more than one million images annotated of the 1000 object categories^58^. We selected 60,000 images from ILSVRC2012 through a three-stage procedure. First, initial images were randomly selected from the database (60 images/category), requiring each image appears square (aspect ratio≈1) and high resolution (>100,000 pixels). Second, the selected images were visually inspected to detect blurred images or wrong category labels. Finally, the improper images detected from the visual inspection were replaced with images that met the criteria required by the above two stages.

#### Stimuli from COCO

The Common Object in Context (COCO) is another large-scale image dataset for computer vision, containing 2.5 million labeled everyday objects across 328,000 images with multiple annotations^40^. Images in COCO show more versatility and more variation than those in ImageNet. The COCO experiment was designed for the precise mapping of brain activity patterns induced by individual images which contain multiple interacting objects we encounter daily. This requires us to present each image at least one time in each run. Considering that a run should ideally be less than ten minutes, 120 images presented once with some randomly inserted blank trials (3 seconds/trial), resulting in a run lasting approximately 8 minutes would be a good choice for us. Further, a three-fold clustering procedure was developed to draw 120 images from COCO that can cover the image space expanded by the COCO images as much as possible and thus be diverse and representative of everyday life. First, the activations from the last layer of a pre-trained ResNet-152^59^ were used to encode each COCO image. Second, a K-means clustering algorithm was used to group these images into 120 clusters. Finally, the image closest to the center of each cluster was selected as the candidate stimuli for our COCO sessions.

#### ImageNet experiment

ImageNet images were presented using a rapid event-related design with a 1-second ON/3-second OFF trial structure (Fig. 1b). We chose the rapid event-related design and the relatively short stimulus duration to maximize the number of trials and minimize eye movements^21,32^. Each session comprises 1000 images (1 image per category), presented evenly in ten runs. First, the stimuli sequence of a session was optimized with Optseq (https://surfer.nmr.mgh.harvard.edu/optseq/) to avoid the semantically similar objects (e.g., 130 categories of dogs) appearing in succession. Second, the optimized stimuli sequence was divided into ten runs evenly. Finally, a blank trial was inserted every five trials. Four blank trials were also added at the beginning and end of each run. Note that none of the images were repeated in any run, any session, and any participant. The stimuli from different sessions and participants were aligned in conditions (i.e., categories), but not in individual images. Specifically, for sub01-sub09, each viewed 4000 unique images (i.e., 1000 images per session), adding up to 36,000 unique images; for sub10–30, each viewed 1000 unique images, totaling 21,000 unique images. As a result, 57,000 unique images from the ImageNet database were used in the ImageNet experiment. Participants were asked to fixate on the dot in the center of the screen and press one of two response buttons as quickly as possible after an image disappeared to indicate that the most salient object presented in the image was an animate or inanimate object. Specifically, they were instructed to press a button with their right thumb for an animate object and press another button with their left thumb for an inanimate object. Participants engage well with the task. The mean recognition accuracy is 83.7% across participants and except for sub-02, all participants show comparable good performance (Fig. 1d).

#### COCO experiment

COCO Images were presented using a rapid event-related design with a 0.5-second ON/2.5-second OFF trial structure (Fig. 1c). Each session is composed of ten runs. In each run, the 120 COCO images were randomly presented once on a gray screen, and 30 blank trials were randomly inserted between images. Four blank trials were also added at the beginning and end of each run. Different from the ImageNet experiment, participants shared the same 120 COCO images which were presented for each run and each session. Participants were asked to detect a color change of the fixation. The color changes exclusively happened in the blank trials with a probability of 50%. The participants perform very well in this task with a mean recognition accuracy as 92%. Only sub-01 shows inferior performance.

#### Functional localizer experiment

A retinotopic mapping task and a category-selective localizer task were conducted in the functional localizer session. Eight runs of the retinotopic mapping adapted from the Human Connectome Project 7 T Retinotopy experiment^50^ were conducted to map the retinotopic organization of the early visual cortex in individual participants. Stimuli consisted of slowly moving apertures filled with a dynamic colorful texture and pink noise background. Apertures and textures were updated at a rate of 15 Hz. Two runs used a rotating wedge (RETCCW and RETCW), two runs used an expanding (RETEXP) or contracting ring (RETCON), and four runs used a moving bar (RETMB).

Four runs of category-selective localizer were performed using fLoc (http://vpnl.stanford.edu/fLoc/) to define subject-specific category-selective areas^51^. The experiment presents grayscale images depicting ten different categories of stimulus, including characters (word and number), bodies (body and limb), faces (adult and child), places (corridor and house), and objects (car and instrument). Each trial consists of eight images from a given category sequentially presented, lasting 4 seconds. Participants performed a one-back task on the sequentially presented images.

### Magnetic resonance imaging acquisition

Magnetic Resonance Imaging (MRI) was conducted on a Siemens 3 Tesla (3 T) MAGNETOM Prisma MRI scanner at the BNU Imaging Center for Brain Research, Beijing, China, using a 64-channel phased-array head coil. Multiple runs of task-based fMRI were acquired in ImageNet, COCO, and functional localizer session. A spin-echo field map was acquired to correct the magnetic field distortion in the middle of each fMRI session. A T1-weighted (T1w) anatomical image was obtained for each participant at the beginning of the first fMRI session. Earplugs were used to attenuate scanner noise, and extendable padded head clamps were used to restrain head motion. No physiological data and eye-tracking data were recorded. As a result, we cannot provide quantitative measures on how participants successfully maintained central fixation though all of the participants orally reported that they were able to fixate on the fixation point.

#### Functional MRI

Blood-oxygenation-level-dependent (BOLD) fMRI data were collected using a gradient-echo, multi-band (MB) accelerated echo-planar imaging T2*-weighted sequence: 72 transversal slices parallel to the AC-PC line; in-plane resolution, 2 × 2 mm; slice thickness, 2 mm without gap; field of view (FOV), 200 × 200 mm; repetition time (TR), 2000 ms; echo time (TE), 34 ms; flip angle, 90°; echo spacing, 0.54 ms; bandwidth, 2380 Hz/Px; and MB factor, 3.

#### Field map

The field map was acquired using a dual-echo gradient echo pulse sequence: 72 slices with the same position as fMRI; slice thickness, 2 mm without gap; voxel size, 2 × 2 × 2 mm; FOV, 200 × 200 mm; TR, 720 ms; TE1/TE2, 4.92/7.38 ms; and flip angle, 60°.

#### Structural MRI

Structural T1w images were acquired for anatomical reference with a three-dimensional magnetization-prepared rapid acquisition gradient echo sequence: 1 slab; 208 sagittal slices; FOV, 256 × 256 mm; slice thickness, 1 mm; isotropic voxel size, 1 × 1 × 1 mm; TR, 2530 ms; TE 2.27 ms; TI, 1100 ms; and flip angle, 7°.

### Data analysis

#### Overall data processing pipeline

**Fig. 2:**
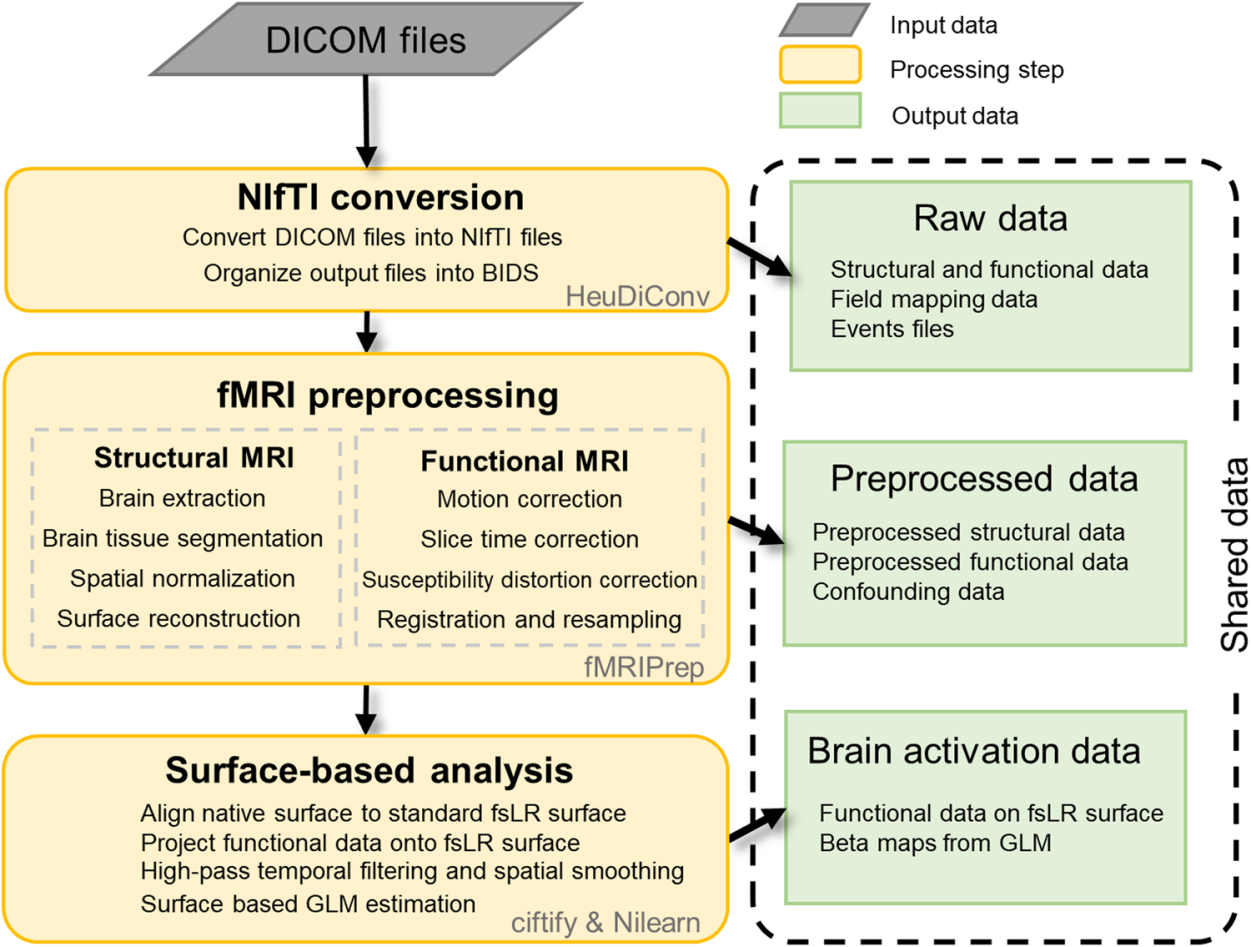
Overview of data processing pipeline and shared data. The raw data were preprocessed with fMRIPrep and projected to the standard fsLR surface with ciftify. The surface-based general linear model (GLM) analysis was performed for data quality checks. Both the raw data and the derived data from preprocessing and GLM are shared.

The Digital Imaging and Communications in Medicine (DICOM) images acquired from the Siemens scanner were converted into the Neuroimaging Informatics Technology Initiative (NIfTI) format and reorganized into the Brain Imaging Data Structure (BIDS)^60^ using HeuDiConv (https://github.com/nipy/heudiconv). The facial features were removed from anatomical T1w images using the PyDeface (https://github.com/poldracklab/pydeface). The data were then preprocessed and analyzed to produce the derived data for sharing and quality validation (Fig. 2).

#### Preprocessing of MRI data

The MRI data were preprocessed by fMRIPrep 20.2.1^61^. Detailed information on fMRIPrep pipelines can be found in the online documentation of the fMRIPrep (https://fmriprep.org). In brief, individual structural MRI was intensity corrected, skull stripped, and normalized to ICBM152 nonlinear asymmetrical template using ANTs^62^. Then, brain tissue was segmented using FAST^63^, and brain surfaces were reconstructed using FreeSurfer^64^. Functional MRI data were motion corrected using MCFLIRT^65^, slice-time corrected using 3dTshift^66^, field-distortion corrected using SDCflows^67^, and co-registered to the T1w using bbregister^68^.

All the preprocessed individual fMRI data were registered onto the 32k fsLR surface space using the ciftify toolbox^69^ and a high-pass temporal filter (cutoff = 128 s) and a spatial smooth (FWHM = 4 mm) were applied to data from each run.

#### GLM analysis of the ImageNet and COCO experiment

For both ImageNet and COCO experiments, we estimated the vertex responses evoked by each image by a surface-based general linear model (GLM) analysis with the Nilearn package^70^. Specifically, functional data from each run and each session were regressed vertex by vertex with a GLM in which the ideal time series induced by each image was modeled as a convolution of its timing function with a canonical hemodynamic response function. Six parameters from motion correction were also included into the model to account for the effect of head movements. The vertex-specific amplitude of the responses (i.e., beta values) estimated for each image was used for further analyses.

#### GLM analysis of category-selective localizer experiment

A GLM analysis was also conducted for the category-selective localizer experiment to define subject-specific category-selective regions. A boxcar kernel convolved with a canonical hemodynamic response function was used to model BOLD signal changes for each of the ten categories of stimulus in each run. Six parameters from motion correction were also included in the model to account for the effect of head movements. The brain activation for each category was computed by a linear contrast between the category and the averaging of other categories. A fixed‐effect analysis was done to combine all runs within each session, and the resulting activation maps were used to define the category-selective region of interest for each participant.

#### Population receptive field analysis of retinotopic mapping experiment

The fMRI data from the retinotopic mapping experiment were analyzed by a population receptive field (pRF) model implemented in the analyzePRF toolbox (http://cvnlab.net/analyzePRF/) to characterize individual retinotopic representation^71,72^. The model predicts fMRI time series as the convolution of the stimulus-related time series and a canonical hemodynamic response function. The stimulus-related time series are in turn generated by computing the dot product between the stimulus apertures and a 2D isotropic Gaussian, scaling and applying a static power-law nonlinearity. Several parameters of interest are produced from the pRF model for each vertex including phase angle, eccentricity, and pRF size.

## Data Records

**Fig. 3:**
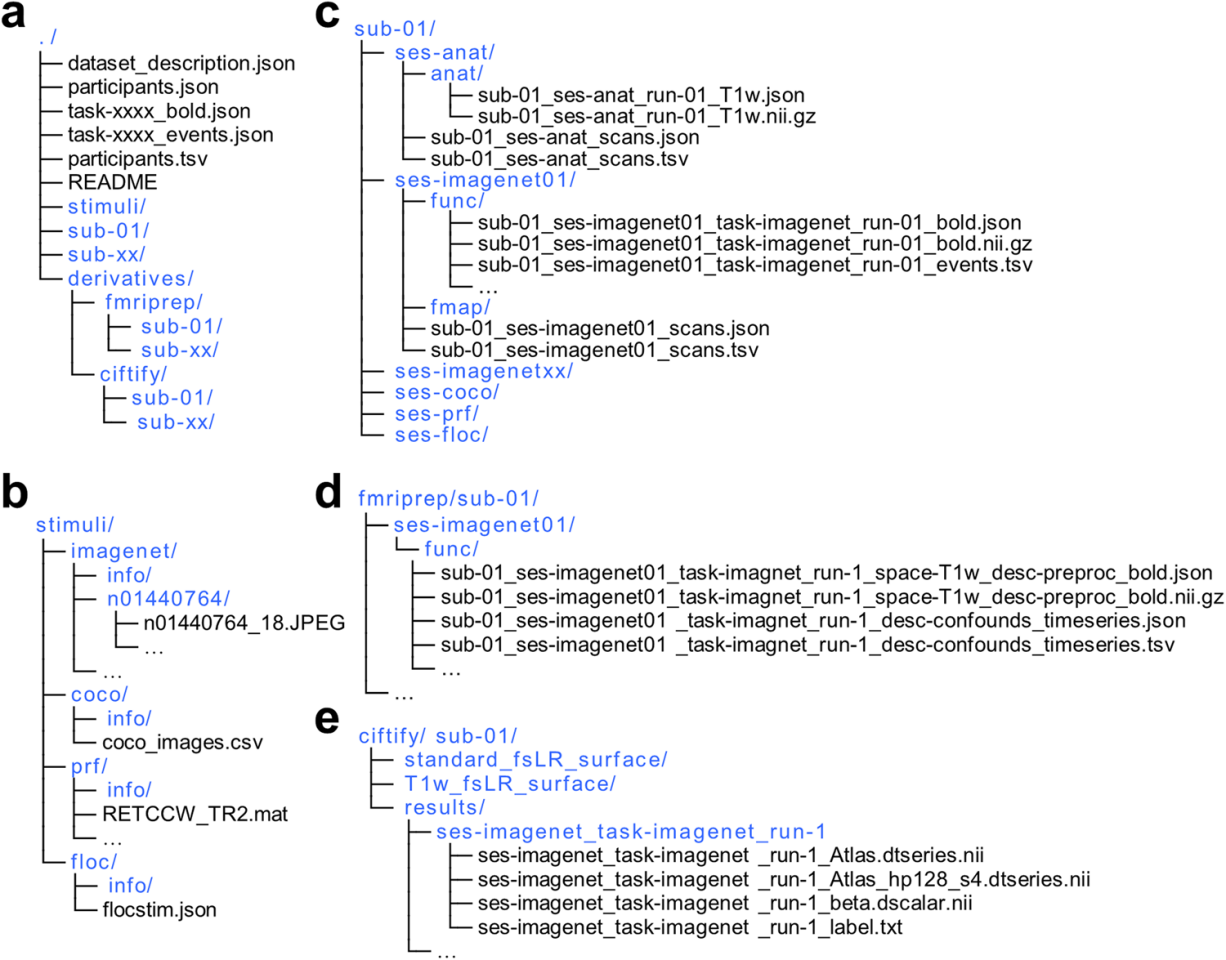
The file structure of Natural Object Dataset (NOD). (**a**) The overall file structure of NOD. (**b**) The file structure of stimulus images. (**c**) The file structure of the raw data from a sample participant. (**d**) The file structure of the preprocessed data from a sample participant. (**e**) The file structure of the derived surface-based data from a sample participant.

The data can be accessed from the OpenNeuro public repository (accession number: ds004496)^73^, organized according to the Brain-Imaging-Data-Structure (BIDS) Specification version 1.7.0. In short, the raw data from each subject are saved in “sub-<ID>” directories; The preprocessed volume data and the derived surface-based data are stored in “derivatives/fmriprep” and “derivatives/ciftify” directories, respectively (Fig. 3a).

### Stimulus images

The stimulus images for different fMRI experiments are deposited in separate folders: “stimuli/imagenet”, “stimuli/coco”, “stimuli/prf”, and “stimuli/floc” (Fig. 3b). First, the images for the ImageNet experiment are stored in 1000 subfolders of the “imagenet” folder. Each subfolder is named by synset id whose further information can be found in the “imagenet/info/synset_words.txt”. Second, the “coco_images.csv” file under the “coco” folder lists the names of images used in the COCO experiment. Finally, the “README.TXT” located in the “info” folder under the corresponding stimulus folder provides more details of the stimuli used in each experiment.

### Raw MRI data

The folder for each participant consists of several session folders: “ses-anat”, “ses-coco”, “ses-imagenet”, “ses-prf”, and “ses-floc”. The session folder in turn includes one or two folders, named “anat”, “func” or “fmap”, for corresponding modality data. The scan information for each session is provided in the “sub-<subID>_ses-<sesID>_scans.tsv” file (Fig. 3c). In the “func” folder, the “sub-<subID>_ses-<sesID>_task-<taskID>_run-<runID>_events.tsv” file contains task events of each run. The stimulus information for each trial is listed in the last column of this events file, which is named “stim_file” and “condition” for the ImageNet and the COCO experiments, respectively. As a result, the specific stimulus image for each trial can be located in the “stimuli” folder according to the stimuli information listed in the last column of the events file.

### Preprocessed volume data from fMRIPrep

The preprocessed volume-based fMRI data are in subject’s native space, saved as “sub-<subID>_ses-<sesID>_task-<taskID>_run-<index>_space-T1w_desc-preproc_bold.nii.gz” for each functional run (Fig. 3d). A “sub-<subID>_ses-<sesID>_task-<taakID>_run-<index>_desc-confounds_timeseries.tsv” file stores the confounds variable extracted by fMRIPrep. Other auxiliary files can also be found under each session folder.

### Preprocessed surface-based data from ciftify

Under each run folder, the preprocessed surface-based data are saved standard fsLR space, named as “sub-<subID>/results/ses-<sesID>_task-<taskID>_run-<index>/ses-<sesID>_task-<taskID>_run-<index>_Atlas.dtseries.nii” for each functional run. The standard and native fsLR surface can be found in the “standard_fsLR_surface” and “T1w_fsLR_surface” folders, respectively (Fig. 3e).

### Brain activation data from surface-based analysis

The brain activation data are derived from GLM analyses on the standard fsLR space, saved as “sub-<subID>/results/ses-<sesID>_task-<taskID>_run-<index>/ses-<sesID>_task-<taskID>_run-<index>_beta.dscalar.nii” for each functional run from both ImageNet and COCO experiments (Fig. 3e). Within each run folder, the auxiliary information about labels or conditions can be found in “ses-<sesID>_task-<taskID>_run-<index>_label.txt”. Additionally, individual-specific character-, faces-, body- and place–selective activation maps and ROIs are stored in “sub-<subID>/results/ses-floc_task-floc” folder; And pRF parameters maps are stored in “sub-<subID>/results/ses-prf_task-prf” folder.

## Technical Validation

### Basic quality control indicates the data show good quality

**Fig. 4:**
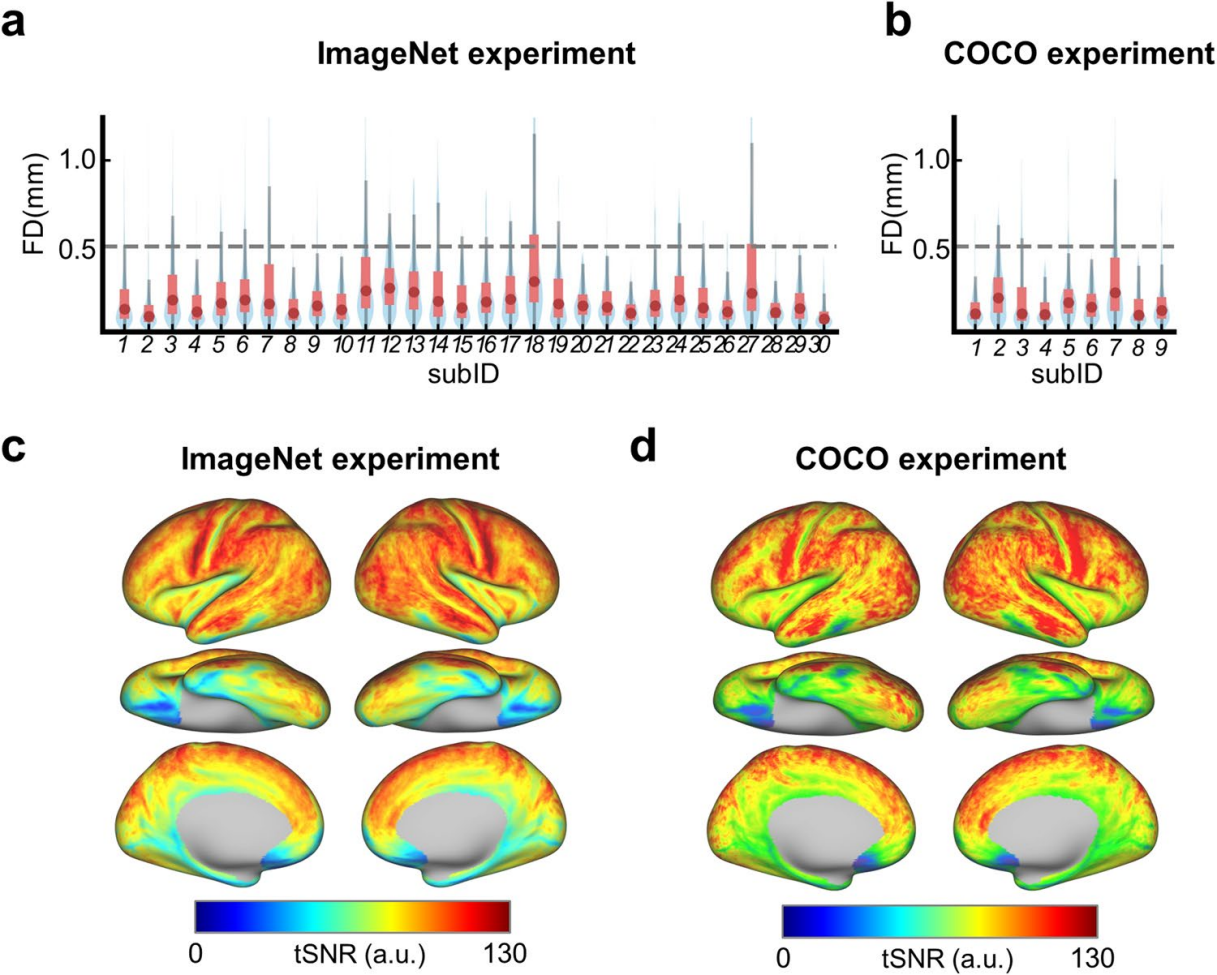
Basic data quality check of the data from the ImageNet and the COCO experiments. (**a**) The violin plots of FD in the ImageNet experiment for each participant. The red dots, pink bars and gray vertical lines indicate the mean, the range of quartiles, and the 95% confidence interval, respectively. (**b**) The violin plots of FD in the COCO experiment. (**c**) The group mean tSNR maps across all participants (N = 30) for the ImageNet experiment. (**d**) The group mean tSNR maps across all participants (N = 9) for the COCO experiment.

The magnitude of participants’ head motion and the temporal signal-to-noise ratio (tSNR) of the fMRI time courses were evaluated for the basic quality control of the NOD data. The head motion of the participants was measured using the framewise displacement (FD) metric, which measures the relative movement of the head from one volume to the next^74^. The FD from each time point was summarized across all runs separately for each participant from both ImageNet and COCO experiments. As shown in Fig. 4, all participants except sub-18 in the ImageNet experiment show a few volumes with FD larger than 0.5 mm, a criterion often used to detect the volume with large head motion^74^. The percentage of time points with high FD (i.e., greater than 0.5 mm) was further quantified. It is revealed that across participants, the median of the percentage of high FD time points is 6.3% for the ImageNet experiment and 5.4% for the COCO experiment. These results indicate that head motion is in good control under both ImageNet and COCO experiments.

The tSNR is a widely used metric for assessing the ability to detect brain activation in fMRI data^75^. Specifically, the tSNR was computed as the mean of each vertex’s time course divided by its standard deviation on the preprocessed data of each run, and then averaged across runs to produce individual tSNR maps for both ImageNet and COCO experiments. The individual tSNR maps were finally averaged to construct the group mean tSNR maps for ImageNet (Fig. 4c) and COCO (Fig. 4d) experiments, separately. The median value of the group mean tSNR across vertices of the whole cortex is 82.72 for the ImageNet experiment and 83.03 for the COCO experiment, respectively. Except that the anterior temporal lobe shows low tSNR due to susceptibility-induced signal loss^76^, visual-related cortex shows high tSNR. Taken together, these results indicate that the data show good tSNR in both experiments.

### The visual cortex shows reliable responses in ImageNet and COCO experiment

**Fig. 5:**
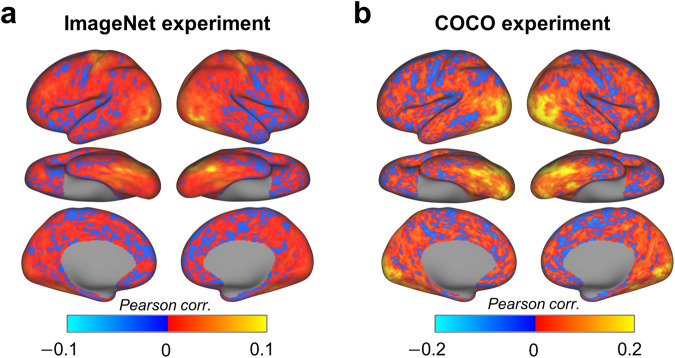
The fMRI data from ImageNet and COCO experiments show higher response reliability in visual areas involved in object recognition. (**a**) The group mean maps of test-retest reliability of BOLD response across 1000 ImageNet categories. The test-retest reliability was first computed with each pair of sessions and then averaged across sessions and participants (N = 9). (**b**) The group mean maps of test-retest reliability of BOLD across 120 COCO images. The test-retest reliability was first calculated between odd runs and even runs within a session (i.e., participant) and averaged across participants (N = 9).

The reliability of the BOLD response evoked by naturalistic stimuli was evaluated for both the ImageNet and COCO experiments. For the ImageNet experiment, nine participants completed four ImageNet sessions in each of which 1000 categories of stimuli were presented. Therefore, we can measure the test-retest reliability of the ImageNet experiment by computing the Pearson correlation between the BOLD responses of the 1000 categories (i.e., beta series) from each pair of sessions within each of the nine participants. As expected, both the lateral occipital cortex and the ventral temporal cortex, which are heavily involved in object processing^8^, present higher test-retest reliability in response to the 1000 categories of naturalistic stimuli than other brain areas (Fig. 5a). Because the participants have reliable behavioral responses (i.e., key pressing) in performing the animacy judgment task on the 1000 categories, the hand motor area appears high reliability. The early visual cortex does not show high reliability because no exemplars of each category are repeatedly presented in different sessions. For the COCO experiment, 120 images were presented in each of the ten runs within a session. We here thus assessed the test-retest reliability of responses to these images by calculating the Pearson correlation between the beta series of 120 images from the odd and even runs on each vertex. As shown in Fig. 5b,most visual areas, including the early visual cortex, ventral temporal cortex, and lateral occipital cortex, show higher test-retest reliability in response to the set of images. In summary, these analyses reveal that fMRI data from ImageNet and COCO experiments show reasonable test-retest reliability across categories and images, respectively, in visual areas involved in object recognition.

### The large-scale topographic animacy map can be revealed with ImageNet experiment at both group and individual levels

**Fig. 6:**
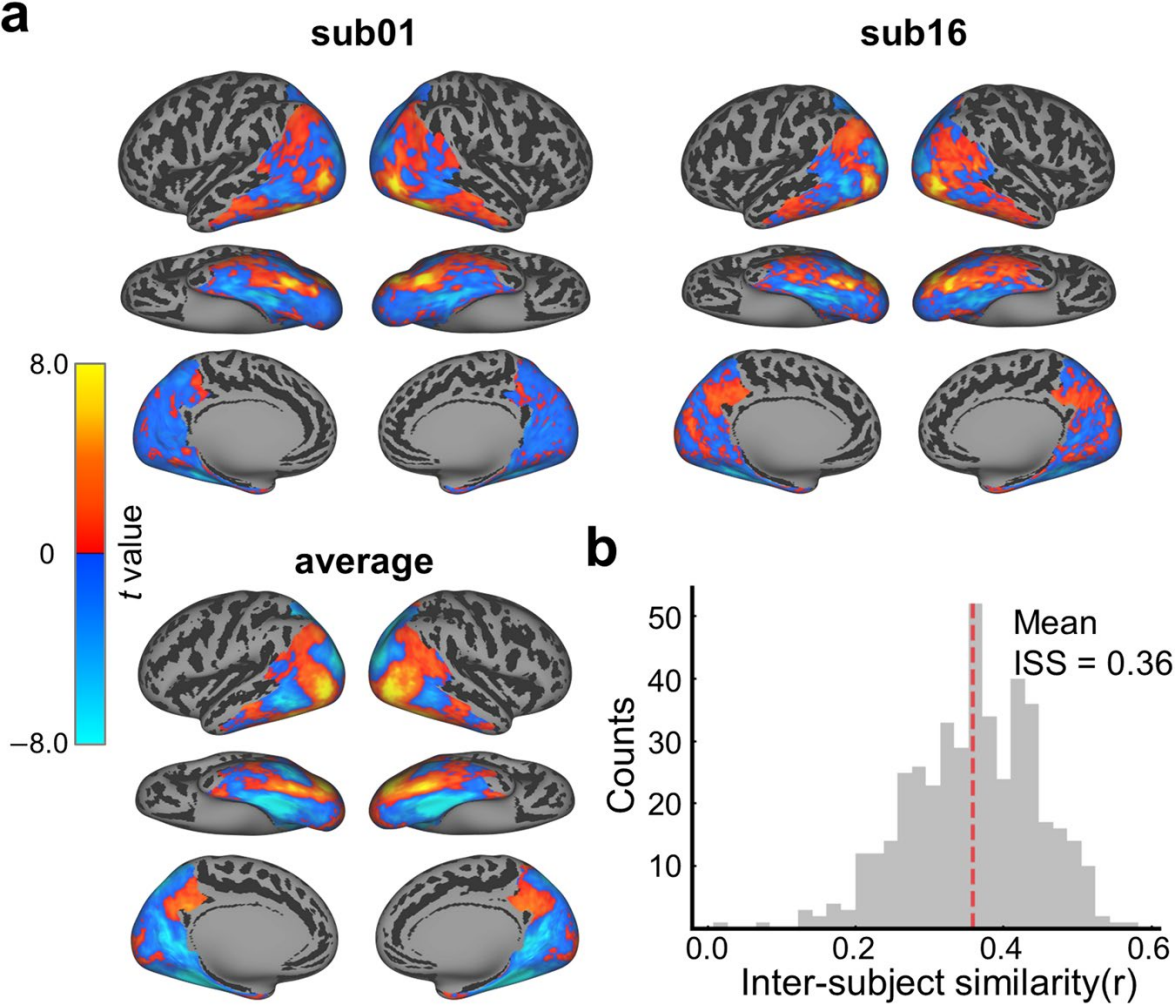
The large-scale individual topographic animacy map revealed with ImageNet fMRI dataset. (**a**) The animacy maps for two sample participants (upper panel) and the group (lower panel). The animacy map was calculated as the t statistics between animate and inanimate conditions. (**b**) The distribution of inter-subject similarity of the animacy maps. The ISS was computed as the Pearson correlation of the animacy maps from each pair of participants.

The existence of a large-scale topographic map for the distinction between animate and inanimate conditions in the lateral to medial visual cortex has been widely documented in the literature^77–79^. We next evaluated whether our fMRI data from a large set of naturalistic stimuli could consistently reveal the animacy map across individuals. Specifically, we contrasted animate versus inanimate conditions from the ImageNet experiment within each of the thirty participants, with a total of 410 animate and 590 inanimate experimental conditions. The statistic maps for the contrast between animate and inanimate conditions were computed with a t-test on each participant. For those participants who completed four ImageNet sessions, only the data from the first session were used. A large-scale topographic animacy map is clearly revealed in individual participants (Fig. 6a upper panel), with the animacy representation organized along the lateral to the medial axis in the ventral pathway and the mid-fusiform sulcus separating the representations for animate and inanimate conditions as shown in many existing studies^77,78^. The group mean of this map is more continuous than that of individuals (Fig. 6a lower panel). The inter-subject similarity (ISS) of the animacy maps was quantitatively measured by computing the Pearson correlation of the animacy maps from each pair of participants. Results show that the distribution of ISS has a mean value of 0.36, indicating the arrangement of the animacy map is well consistent across individuals (Fig. 6b).

### Subject-specific functional regions of interest can be successfully defined with functional localizer experiments

**Fig. 7:**
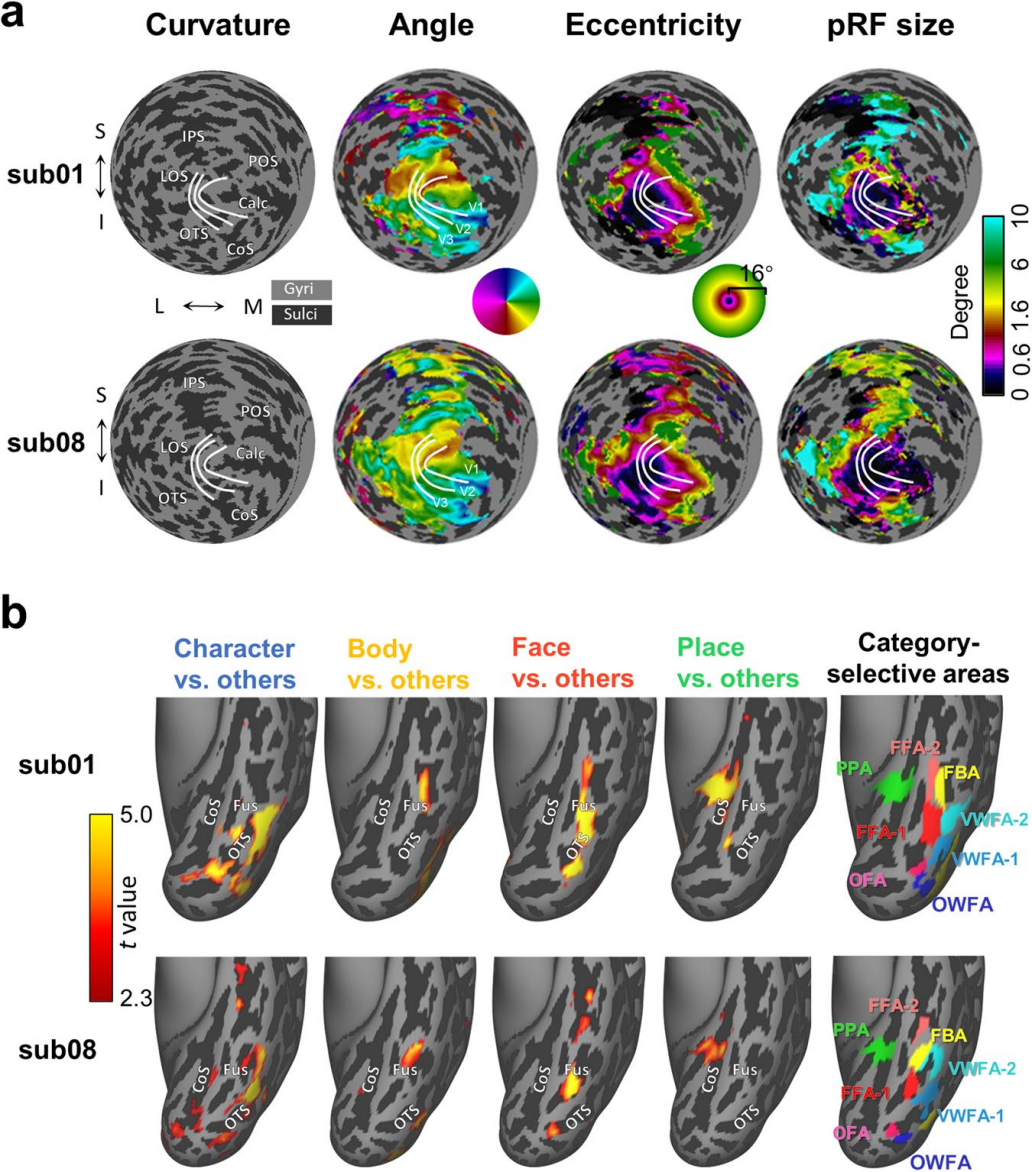
The individual low- and high-level functional areas defined with functional localizer dataset from two representative participants. (**a**) The individual curvature and retinotopic maps on fsLR space of two sample subjects. The first column shows the thresholded curvature on which boundaries of V1, V2, and V3 are indicated as white lines. The second to fourth columns show the angle, eccentricity, and pRF size map derived from individual retinotopic mapping localizer displayed on fsLR sphere space with the subject-specific curvature as underlying. These parametric maps are thresholded respectively at 3.9% (for sub01) and 1.5% (for sub08) variance explained by the pRF models. Labels: S, superior; I, inferior; M, medial; L, lateral; IPS, intraparietal sulcus; LOS, lateral occipital sulcus; POS, parieto-occipital sulcus; Calc, calcarine sulcus; OTS, occipitotemporal sulcus; CoS, collateral sulcus. (**b**) The individual character-, faces-, body- and place-selective activation maps (threshold, t > 2.3, uncorrected) and manually delineated category-selective areas are displayed on the fsLR surface for two example subjects, the upper panel is for sub01 and the lower panel is for sub08. Only the data from the left hemisphere are displayed for saving space. Labels: Fus, fusiform gyrus; OTS, occipitotemporal sulcus; CoS, collateral sulcus; PPA, parahippocampal place area; FFA, fusiform face area; OFA, occipital face area; FBA, fusiform body area; VWFA, visual word form area; OWFA, occipital word form area.

To support fine-scale subject-specific analyses, a retinotopic mapping task and a category-selective localizer task were performed in the functional localizer session of NOD. The fMRI data from the retinotopic mapping were used to map the retinotopic representation of low-level visual areas in individual participants. As shown in Fig. 7a, the polar angle, eccentricity, and pRF size derived from the pRF model analysis on individual data show spatial patterns highly like those derived from the Human Connectome Project 7 T Retinotopy Dataset^50^. In contrast to retinotopic mapping, which aimed to map low-level visual areas, the category-selective localizer is conducted to define high-level areas that are selective for categories. It is revealed that individual functional areas selective to character, face, body, and place can be easily identified with the category-selective localizer^51^ (Fig. 7b). Overall, these analyses demonstrate that the functional localizer of NOD is of good quality in defining subject-specific visual areas.

### Encoding model reveals the hierarchical correspondences between the brain and the deep convolutional neural network

**Fig. 8:**
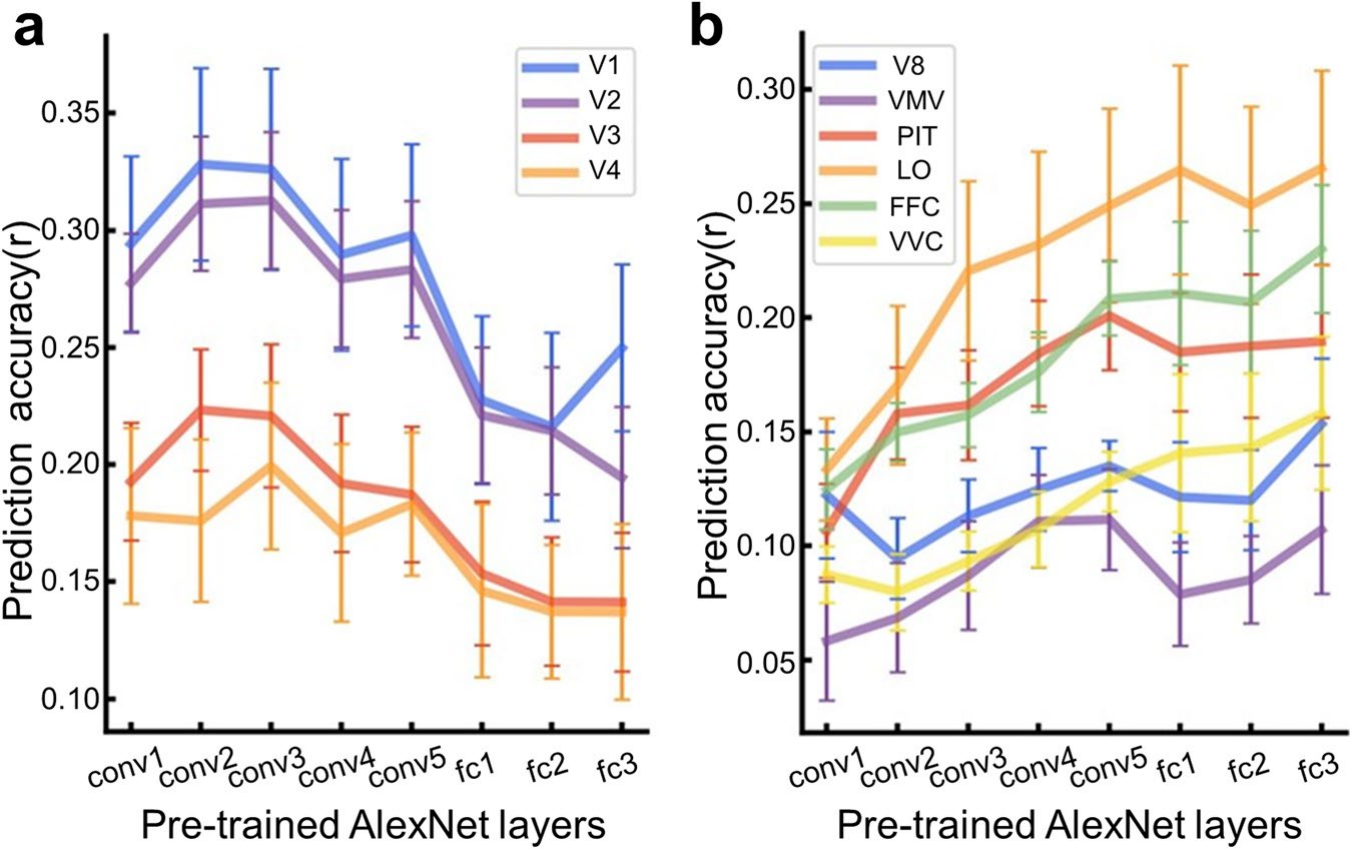
The hierarchical correspondences between the ventral visual pathway and the deep convolutional neural network revealed by encoding models. The ventral visual pathway consists of V1, V2, V3, V4, V8, VMV (ventromedial visual areas), PIT (posterior inferotemporal complex), LO (lateral occipital), FFC (fusiform face complex), and VVC (ventral visual complex) as defined in the multimodal parcellation atlas. (**a**) The prediction performance of the encoding models for low visual areas. (**b**) The prediction performance of the encoding models for high visual areas. The error bar is the standard error of the mean across nine subjects.

Numerous studies have discovered a hierarchical correspondence in representation between deep convolutional neural network (DCNN) and the ventral visual stream in both humans and monkeys^27,52,80,81^. Here, we combined the data from ImageNet, COCO, and functional localizer experiments to build an encoding model to replicate the hierarchical correspondence of representations between the brain and the DCNN. The encoding models were built to map artificial representations from each layer of the pre-trained AlexNet^82^ to neural representations from each area of the ventral visual pathway. The ventral-stream ROIs were defined according to the Human Connectome Project Multi-Modal Parcellation (HCP-MMP), including V1, V2, V3, V4, V8, VMV (ventromedial visual areas), PIT (posterior inferotemporal complex), LO (lateral occipital), FFC (fusiform face complex), and VVC (ventral visual complex)^83^. In order to conserve computational resources, the top 50 vertices, which showed the best model performance in fitting the pRF model to the retinotopic mapping data, were selected from each area to represent the area, and encoding models were then constructed for them. First, the pRF estimated from the retinotopic mapping data was used as a spatial kernel for the vertex to weigh the feature maps from AlexNet to generate the weighted features of each convolutional layer. Second, a linear GLM encoding model with L1 regularization was built for each vertex using the pRF-weighted AlexNet layer features as input. The ImageNet experiment data were used for model training and a four-fold cross-validation framework for hyperparameter tuning. Finally, the prediction accuracies of encoding models were evaluated as the correlation between the predicted and actual responses from the COCO experiment. As depicted in Fig. 8a, the encoding precision of low-level visual regions decreases gradually as the AlexNet layers ascend. In contrast, the encoding accuracy of the high-level visual areas shows opposite trends (Fig. 8b). In conclusion, our data confirm an early-to-early and late-to-late correspondence pattern between the ventral visual stream and DCNN layers.

## Usage Notes

The dataset provides unique opportunities for our understanding of the visual processing of naturalistic stimuli. First, given the abundance of naturalistic images with BOLD responses recorded, NOD will be useful for fine-grained exploration into the neural representation of a wide range of visual features and semantics. Importantly, compared to other publicly available datasets, NOD achieves a good balance between sampling variation across individuals and sampling variation across stimuli. On one hand, each of the 30 participants completed an ImageNet scan session with 1,000 ImageNet categories to sample variation across individuals as much as possible. The large sample size of subjects could support us to draw generalizable claims on the category-selective activity patterns from the population. On the other hand, nine of 30 participants completed three additional ImageNet sessions with different images to sample variation across stimuli as much as possible within an individual. This enables us to robustly characterize the category-selective activity patterns across different sets of stimuli within an individual. Second, the data from the COCO session, in which 120 COCO images were presented ten times, allows for precise mapping of brain activity patterns for individual images and investigating stimulus-independent effects such as neural variability and noise correlation^84,85^. Furthermore, the relatively large stimulus size (16° wide) compared to other publicly available datasets (usually ~ 8°) enables users to examine neural representation of more peripheral stimuli. Finally, when combined with other large-scale datasets such as BOLD5000, NSD, and THINGS, NOD can contribute to bridging the disciplines of visual neuroscience and artificial intelligence by being used as biological constraints to create deep neural network models that more closely resemble the human visual system^32,86,87^.

While we believe that this dataset is a unique resource for human visual neuroscience, we should acknowledge its limitations. First, as noted above, no image was repeated in the ImageNet session, which may lead to noisy estimates for the BOLD response of a single image. Although our analyses proved those estimates were still sufficient for uncovering some general effects, we would like to point out that a more effective strategy is to construct the GLM on the feature level instead of the image level to reveal fine-scale effects. As each image is composed of multiple features, constructing the GLM on the feature level can effectively improve the statistical power. Besides, the advanced denoising toolboxes such as GLMdenoise^88^ and GLMsingle^89^ could be used to improve the accuracy of BOLD response estimates for the NOD data. Second, although a rapid event-related fMRI paradigm was used, sluggish fMRI signals are incapable of resolving neural dynamics at the milliseconds level. For this, we are conducting a MEG experiment with the same participants and the same stimuli as NOD to record the dynamic responses for visual processing of naturalistic stimuli. We hope this will further complement NOD with MEG measurements to resolve the spatiotemporal dynamics of visual processing^33,90^.

## Data Availability

All codes for the experimental design, data organization, and technique validation are available at https://github.com/BNUCNL/NOD-fmri. Preprocessing was performed using fMRIPrep version 20.2.1 (https://fmriprep.org). Grayordinate-based (CIFTI format) brain activation analysis was performed by combining the Ciftify (https://github.com/edickie/ciftify) and HCP pipelines (https://github.com/Washington-University/HCPpipelines).

## References

[1] Ringach DL (2004). Mapping receptive fields in primary visual cortex. J. Physiol..

[2] Grill-Spector K, Malach R (2004). The human visual cortex. Annu. Rev. Neurosci..

[3] Carandini M (2005). Do we know what the early visual system does?. J. Neurosci..

[4] Felsen G, Dan Y (2005). A natural approach to studying vision. Nat. Neurosci..

[5] Rust NC, Movshon JA (2005). In praise of artifice. Nat. Neurosci..

[6] Kanwisher N (2010). Functional specificity in the human brain: a window into the functional architecture of the mind. Proc. Natl. Acad. Sci..

[7] Graham NV (2011). Beyond multiple pattern analyzers modeled as linear filters (as classical V1 simple cells): Useful additions of the last 25 years. Vision Res..

[8] Grill-Spector K, Weiner KS (2014). The functional architecture of the ventral temporal cortex and its role in categorization. Nat. Rev. Neurosci..

[9] Pasupathy A, Popovkina DV, Kim T (2020). Visual functions of primate area V4. Annu. Rev. Vis. Sci..

[10] Arcaro MJ, Livingstone MS (2021). On the relationship between maps and domains in inferotemporal cortex. Nat. Rev. Neurosci..

[11] Touryan J (2001). Analysis of sensory coding with complex stimuli. Curr. Opin. Neurobiol..

[12] Kayser C (2004). Processing of complex stimuli and natural scenes in the visual cortex. Curr. Opin. Neurobiol..

[13] Calhoun VD, Pearlson GD (2012). A selective review of simulated driving studies: combining naturalistic and hybrid paradigms, analysis approaches, and future directions. NeuroImage.

[14] Turner MH, Sanchez Giraldo LG, Schwartz O, Rieke F (2019). Stimulus- and goal-oriented frameworks for understanding natural vision. Nat. Neurosci..

[15] Naselaris T, Allen E, Kay K (2021). Extensive sampling for complete models of individual brains. Curr. Opin. Behav. Sci..

[16] Sonkusare S, Breakspear M, Guo C (2019). Naturalistic stimuli in neuroscience: critically acclaimed. Trends Cogn. Sci..

[17] DuPre E, Hanke M, Poline J-B (2020). Nature abhors a paywall: how open science can realize the potential of naturalistic stimuli. NeuroImage.

[18] Jääskeläinen IP, Sams M, Glerean E, Ahveninen J (2021). Movies and narratives as naturalistic stimuli in neuroimaging. NeuroImage.

[19] Zhang Y, Kim J-H, Brang D, Liu Z (2021). Naturalistic stimuli: a paradigm for multiscale functional characterization of the human brain. Curr. Opin. Biomed. Eng..

[20] Kringelbach ML, Perl YS, Tagliazucchi E, Deco G (2023). Toward naturalistic neuroscience: mechanisms underlying the flattening of brain hierarchy in movie-watching compared to rest and task. Sci. Adv..

[21] Kay KN, Naselaris T, Prenger RJ, Gallant JL (2008). Identifying natural images from human brain activity. Nature.

[22] Haxby JV (2011). A common, high-dimensional model of the representational space in human ventral temporal cortex. Neuron.

[23] Hanke M (2014). A high-resolution 7-tesla fMRI dataset from complex natural stimulation with an audio movie. Sci. Data.

[24] Hanke M (2016). A studyforrest extension, simultaneous fMRI and eye gaze recordings during prolonged natural stimulation. Sci. Data.

[25] Wen H (2018). Neural encoding and decoding with deep learning for dynamic natural vision. Cereb. Cortex.

[26] Aliko S, Huang J, Gheorghiu F, Meliss S, Skipper JI (2020). A naturalistic neuroimaging database for understanding the brain using ecological stimuli. Sci. Data.

[27] Visconti di Oleggio Castello M, Chauhan V, Jiahui G, Gobbini MI (2020). An fMRI dataset in response to “The Grand Budapest Hotel”, a socially-rich, naturalistic movie. Sci. Data.

[28] Lee H, Chen J, Hasson U (2023). A functional neuroimaging dataset acquired during naturalistic movie watching and narrated recall of a series of short cinematic films. Data Brief.

[29] Alexander LM (2017). An open resource for transdiagnostic research in pediatric mental health and learning disorders. Sci. Data.

[30] Nastase, S. A., Halchenko, Y. O., Connolly, A. C., Gobbini, M. I. & Haxby, J. V. Neural responses to naturalistic clips of behaving animals in two different task contexts. *Front. Neurosci*. **12**, (2018). 10.3389/fnins.2018.00316 PMC5962655 29867327

[31] Chang N (2019). BOLD5000, a public fMRI dataset while viewing 5000 visual images. Sci. Data.

[32] Allen EJ (2022). A massive 7T fMRI dataset to bridge cognitive neuroscience and artificial intelligence. Nat. Neurosci..

[33] Hebart, M. N. *et al.* THINGS-data, a multimodal collection of large-scale datasets for investigating object representations in human brain and behavior. *eLife* **12**, e82580 (2023). 10.7554/eLife.82580 PMC10038662 36847339

[34] Agtzidis I, Meyhöfer I, Dorr M, Lencer R (2020). Following Forrest Gump: smooth pursuit related brain activation during free movie viewing. NeuroImage.

[35] Li L, Lu B, Yan C-G (2020). Stability of dynamic functional architecture differs between brain networks and states. NeuroImage.

[36] Visconti di Oleggio Castello M, Haxby JV, Gobbini MI (2021). Shared neural codes for visual and semantic information about familiar faces in a common representational space. Proc. Natl. Acad. Sci..

[37] Kumar S, Ellis CT, O’Connell TP, Chun MM, Turk-Browne NB (2020). Searching through functional space reveals distributed visual, auditory, and semantic coding in the human brain. PLOS Comput. Biol..

[38] Wang C (2022). Reconstructing rapid natural vision with fMRI-conditional video generative adversarial network. Cereb. Cortex.

[39] Deng, J. *et al*. ImageNet: a large-scale hierarchical image database. In *2009 IEEE Conference on Computer Vision and Pattern Recognition* 248–255 (2009).

[40] Lin, T.-Y. *et al*. Microsoft COCO: common objects in context. In *Computer Vision – ECCV 2014* (eds. Fleet, D., Pajdla, T., Schiele, B. & Tuytelaars, T.) 740–755 (2014).

[41] Xiao, J., Hays, J., Ehinger, K. A., Oliva, A. & Torralba, A. SUN database: large-scale scene recognition from abbey to zoo. In *2010 IEEE Computer Society Conference on Computer Vision and Pattern Recognition* 3485–3492 (2010).

[42] Hebart MN (2019). THINGS: a database of 1,854 object concepts and more than 26,000 naturalistic object images. PLOS ONE.

[43] Sexton, N. J. & Love, B. C. Reassessing hierarchical correspondences between brain and deep networks through direct interface. *Sci. Adv*. (2022). 10.1126/sciadv.abm2219 PMC9278854 35857493

[44] Bannert MM, Bartels A (2022). Visual cortex: big data analysis uncovers food specificity. Curr. Biol..

[45] Skyberg, R., Tanabe, S., Chen, H. & Cang, J. Coarse-to-fine processing drives the efficient coding of natural scenes in mouse visual cortex. *Cell Rep*. **38**, (2022). 10.1016/j.celrep.2022.110606 PMC9189856 35354030

[46] Roth ZN, Kay K, Merriam EP (2022). Natural scene sampling reveals reliable coarse-scale orientation tuning in human V1. Nat. Commun..

[47] Khosla M, Ratan Murty NA, Kanwisher N (2022). A highly selective response to food in human visual cortex revealed by hypothesis-free voxel decomposition. Curr. Biol..

[48] Kurzawski JW (2022). Short-term plasticity in the human visual thalamus. eLife.

[49] Pennock IML (2023). Color-biased regions in the ventral visual pathway are food selective. Curr. Biol..

[50] Benson NC (2018). The human connectome project 7 tesla retinotopy dataset: description and population receptive field analysis. J. Vis..

[51] Stigliani A, Weiner KS, Grill-Spector K (2015). Temporal processing capacity in high-level visual cortex is domain specific. J. Neurosci..

[52] Yamins DLK (2014). Performance-optimized hierarchical models predict neural responses in higher visual cortex. Proc. Natl. Acad. Sci..

[53] Kriegeskorte N (2015). Deep neural networks: a new framework for modeling biological vision and brain information processing. Annu. Rev. Vis. Sci..

[54] Khosla M, Ngo GH, Jamison K, Kuceyeski A, Sabuncu MR (2021). Cortical response to naturalistic stimuli is largely predictable with deep neural networks. Sci. Adv..

[55] Storrs KR, Kietzmann TC, Walther A, Mehrer J, Kriegeskorte N (2021). Diverse deep neural networks all predict human inferior temporal cortex well, after training and fitting. J. Cogn. Neurosci..

[56] Brainard DH (1997). The Psychophysics Toolbox. Spat. Vis..

[57] Miller GA (1995). WordNet: a lexical database for English. Commun. ACM.

[58] Russakovsky O (2015). ImageNet large scale visual recognition challenge. Int. J. Comput. Vis..

[59] He, K., Zhang, X., Ren, S. & Sun, J. Deep residual learning for image recognition. In *Proc. IEEE Conference on Computer Vision and Pattern Recognition* 770–778 (2016).

[60] Gorgolewski KJ (2016). The brain imaging data structure, a format for organizing and describing outputs of neuroimaging experiments. Sci. Data.

[61] Esteban O (2019). fMRIPrep: a robust preprocessing pipeline for functional MRI. Nat. Methods.

[62] Avants BB, Epstein CL, Grossman M, Gee JC (2008). Symmetric diffeomorphic image registration with cross-correlation: evaluating automated labeling of elderly and neurodegenerative brain. Med. Image Anal..

[63] Zhang Y, Brady M, Smith S (2001). Segmentation of brain MR images through a hidden Markov random field model and the expectation-maximization algorithm. IEEE Trans. Med. Imaging.

[64] Fischl B (2012). FreeSurfer. NeuroImage.

[65] Jenkinson M, Bannister P, Brady M, Smith S (2002). Improved optimization for the robust and accurate linear registration and motion correction of brain images. NeuroImage.

[66] Cox RW, Hyde JS (1997). Software tools for analysis and visualization of fMRI data. NMR in Biomedicine.

[67] Esteban O, Goncalves M, Markiewicz CJ (2022). Zenodo.

[68] Greve DN, Fischl B (2009). Accurate and robust brain image alignment using boundary-based registration. NeuroImage.

[69] Dickie EW (2019). Ciftify: A framework for surface-based analysis of legacy MR acquisitions. NeuroImage.

[70] Abraham, A. *et al*. Machine learning for neuroimaging with scikit-learn. *Front. Neuroinformatics* **8**, (2014). 10.3389/fninf.2014.00014 PMC3930868 24600388

[71] Dumoulin SO, Wandell BA (2008). Population receptive field estimates in human visual cortex. NeuroImage.

[72] Kay KN, Winawer J, Mezer A, Wandell BA (2013). Compressive spatial summation in human visual cortex. J. Neurophysiol..

[73] Gong Z (2023). OpenNeuro.

[74] Power JD, Barnes KA, Snyder AZ, Schlaggar BL, Petersen SE (2012). Spurious but systematic correlations in functional connectivity MRI networks arise from subject motion. NeuroImage.

[75] Welvaert M, Rosseel Y (2013). On the Definition of Signal-To-Noise Ratio and Contrast-To-Noise Ratio for fMRI Data. PLOS ONE.

[76] Wong C, Gallate J (2012). The function of the anterior temporal lobe: a review of the empirical evidence. Brain Research.

[77] Konkle T, Caramazza A (2013). Tripartite organization of the ventral stream by animacy and object size. J. Neurosci..

[78] Sha L (2015). The animacy continuum in the human ventral vision pathway. J. Cogn. Neurosci..

[79] Conway BR (2018). The organization and operation of inferior temporal cortex. Annu. Rev. Vis. Sci..

[80] Güçlü U, van Gerven MAJ (2015). Deep neural networks reveal a gradient in the complexity of neural representations across the ventral stream. J. Neurosci..

[81] Lindsay GW (2021). Convolutional neural networks as a model of the visual system: past, present, and future. J. Cogn. Neurosci..

[82] Krizhevsky A, Sutskever I, Hinton GE (2017). ImageNet classification with deep convolutional neural networks. Commun. ACM.

[83] Glasser MF (2016). A multi-modal parcellation of human cerebral cortex. Nature.

[84] Zhang R-Y, Wei X-X, Kay K (2020). Understanding multivariate brain activity: evaluating the effect of voxelwise noise correlations on population codes in functional magnetic resonance imaging. PLOS Comput. Biol..

[85] Sokoloski S, Aschner A, Coen-Cagli R (2021). Modelling the neural code in large populations of correlated neurons. eLife.

[86] McClure, P. & Kriegeskorte, N. Representational distance learning for deep neural networks. *Front. Comput. Neurosci*. **10**, (2016). 10.3389/fncom.2016.00131 PMC5187453 28082889

[87] Fong RC, Scheirer WJ, Cox DD (2018). Using human brain activity to guide machine learning. Sci. Rep..

[88] Kay, K., Rokem, A., Winawer, J., Dougherty, R. & Wandell, B. GLMdenoise: a fast, automated technique for denoising task-based fMRI data. *Frontiers in Neuroscience* **7**, (2013). 10.3389/fnins.2013.00247 PMC3865440 24381539

[89] Prince JS (2022). Improving the accuracy of single-trial fMRI response estimates using GLMsingle. eLife.

[90] Cichy RM, Pantazis D, Oliva A (2014). Resolving human object recognition in space and time. Nat. Neurosci..

